# Digitalised remote-delivery of AVATr Simulation in Psychiatry: a unique success in COVID-19 pandemic

**DOI:** 10.1192/bjo.2021.411

**Published:** 2021-06-18

**Authors:** Vimal Mannali, Paul Strickland, Craig laBuscagne, Joy Clift

**Affiliations:** 1 Surrey and Borders Partnership NHS Foundation Trust; 2 Xenodu Virtual Environments

## Abstract

**Aims:**

Surrey and Borders NHS Foundation Trust's AVATr (Augmented Virtual-reality Avatar in Training) is a unique ground-breaking Virtual Patient Simulation System, which uses the Xenodu platform to train learners in essential clinical and complex communication skills. Over 30 patient scenarios have been developed after identifying learner-specific development needs, including exploration of overt psychosis, assessment of capacity, sharing bad news, and neglect in care home residents. Actors are filmed responding to several domains of clinical questions, further categorised into three narrative-modes of being ‘Engaged, Neutral or Disengaged’, to build a bank of scenarios. During the session, the trainee is projected on to a large screen, using a camera and video special effects, which results in a life-like interaction with the Virtual Patient. Trainees can view themselves interacting with the Virtual Patient in real-time, from a unique ‘out-of-body' perspective, immersed in a custom-designed interactive virtual environment. The simulation facilitator engages with the learner and determines the appropriate choices of responses for the Virtual Patient and if needed, can prompt with explorative cues to continue the narrative-linked conversation. AVATr model pioneered in United Kingdom the use of an innovative ‘self-observational approach’ in Psychiatry training. This is different to a first-person perspective used in virtual or augmented-reality systems in several clinical specialties. The use of Facilitated-Debrief and Peer-Debrief in sessions, render another layer to the simulation experience.

**Method:**

During the COVID-19 pandemic, we evolved the AVATr model to remote or hybrid sessions, where simulations were digitally enhanced, and have been run through Microsoft Teams. The simulation facilitator is connected to a multi-user video call, enabling the Virtual Patient to be projected as an attendee using Microsoft Teams.

**Result:**

The hybrid model of AVATr has received tremendous feedback, as it now simulates video-consultations that a vast majority of Psychiatry trainees, especially community-based, undertake due to COVID-19 restrictions. The format of AVATr simulation sessions has remained unchanged, and the remote delivery has been particularly successful as it allows trainees to log in from different remote locations to come together for an interactive training session, without any physical restrictions.

**Conclusion:**

Since 2015, our simulation platform has been utilised for Post-Graduate Medical Education, to enhance essential professional skills and stimulate professional growth. Currently the hybrid model of AVATr is being expanded to Nursing, Psychology and Allied Health Professional (AHP) clinical training streams, along with Undergraduate Medical Education, to address identified gaps in face-to-face training amidst COVID-19 pandemic.

